# Supplementary material for: A high-resolution mRNA expression time course of embryonic development in zebrafish
Source: eLife. 2017 Nov 16;6:e30860. doi: 10.7554/eLife.30860 (PMC5690287; doi:10.7554/eLife.30860)
Supplement: Supplementary file 6. [file elife-30860-supp6.zip › biolayout-clusters-files/Cluster200-genes.html]

Cluster200


# Cluster200: Genes

| Ensembl ID | Gene Name | Chr | Start | End | Biotype |
| --- | --- | --- | --- | --- | --- |
| ENSDARG00000086098 | ERRFI1 (1 of many) | 23 | 20909897 | 20917999 | protein\_coding |
| ENSDARG00000101079 | HECTD4 | 7 | 72168935 | 72278814 | protein\_coding |
| ENSDARG00000073744 | ip6k1 | 22 | 39081803 | 39095132 | protein\_coding |
| ENSDARG00000037783 | proza | 3 | 62146031 | 62156823 | protein\_coding |
| ENSDARG00000090170 | rab11fip4a | 3 | 35893748 | 35986003 | protein\_coding |
| ENSDARG00000061551 | rap1gap2a | 15 | 29309321 | 29384191 | protein\_coding |
| ENSDARG00000021443 | zfp36l1b | 20 | 9588134 | 9593206 | protein\_coding |
